# Estimation of Whole Plant Photosynthetic Rate of Irwin Mango under Artificial and Natural Lights Using a Three-Dimensional Plant Model and Ray-Tracing

**DOI:** 10.3390/ijms19010152

**Published:** 2018-01-04

**Authors:** Dae Ho Jung, Joon Woo Lee, Woo Hyun Kang, In Ha Hwang, Jung Eek Son

**Affiliations:** Department of Plant Science and Research Institute of Agriculture and Life Sciences, Seoul National University, Seoul 08826, Korea; apparition@snu.ac.kr (D.H.J.); jwee2@hanmail.net (J.W.L.); flatengine@hanmail.net (W.H.K.); dlsgk1003@snu.ac.kr (I.H.H.)

**Keywords:** CO_2_ consumption, Irwin mango, light interception, photosynthetic rate model, three-dimensional plant model

## Abstract

Photosynthesis is an important physiological response for determination of CO_2_ fertilization in greenhouses and estimation of crop growth. In order to estimate the whole plant photosynthetic rate, it is necessary to investigate how light interception by crops changes with environmental and morphological factors. The objectives of this study were to analyze plant light interception using a three-dimensional (3D) plant model and ray-tracing, determine the spatial distribution of the photosynthetic rate, and estimate the whole plant photosynthetic rate of Irwin mango (*Mangifera indica* L. cv. Irwin) grown in greenhouses. In the case of mangoes, it is difficult to measure actual light interception at the canopy level due to their vase shape. A two-year-old Irwin mango tree was used to measure the whole plant photosynthetic rate. Light interception and whole plant photosynthetic rate were measured under artificial and natural light conditions using a closed chamber (1 × 1 × 2 m). A 3D plant model was constructed and ray-tracing simulation was conducted for calculating the photosynthetic rate with a two-variable leaf photosynthetic rate model of the plant. Under artificial light, the estimated photosynthetic rate increased from 2.0 to 2.9 μmolCO_2_·m^−2^·s^−1^ with increasing CO_2_ concentration. On the other hand, under natural light, the photosynthetic rate increased from 0.2 μmolCO_2_·m^−2^·s^−1^ at 06:00 to a maximum of 7.3 μmolCO_2_·m^−2^·s^−1^ at 09:00, then gradually decreased to −1.0 μmolCO_2_·m^−2^·s^−1^ at 18:00. In validation, simulation results showed good agreement with measured results with *R*^2^ = 0.79 and RMSE = 0.263. The results suggest that this method could accurately estimate the whole plant photosynthetic rate and be useful for pruning and adequate CO_2_ fertilization.

## 1. Introduction

Photosynthesis of crops grown in greenhouses is an important physiological indicator that determines CO_2_ fertilization and estimates crop growth. The cumulative amount of assimilation products produced as a result of photosynthesis is closely related to crop yield. Thus, the photosynthetic rate of crops can be used for predicting crop production [1] and determining CO_2_ supply [2,3,4]. In general, the photosynthesis of crops grown in greenhouses depends on environmental factors such as light intensity, temperature, CO_2_ concentration, and relative humidity inside the greenhouse. These environmental factors are closely related to the structural characteristics of the greenhouse and the meteorological conditions outside the greenhouse. In particular, the intercepted light intensity on leaves is dependent not only on the environmental factors but also on the morphological characteristics of the crops [5,6]. Other environmental variables, such as light direction, ratio of diffuse light, plant growth stage, and plant density, also affect light interception inside the crop canopy [7,8,9]. Therefore, in order to estimate the whole plant photosynthetic rate of crops, it is important to investigate the light interception of crops related with these environmental and morphological factors.

Mango (*Mangifera indica* L.) is one of the 30 most important crops in the world and is grown mainly in tropical or subtropical regions. Recently, mango cultivation in greenhouses has started in Northeast Asia including Korea and Japan. Due to the spatial constraints within a greenhouse, mango trees are cultivated in a vase shape with a low height through pruning of branches [10]. When cultivated in a vase-shape, a mango tree grows more than two m in height and thus the intercepted light intensities at the top and bottom leaves in the canopy are different. However, it is difficult to measure the actual light interception of a plant canopy due to technical limitations.

Previous studies have therefore estimated whole plant photosynthesis by various modelling approaches. The Farquhar, von Caemmerer and Berry (FvCB) model has been widely used as a single-leaf model because it can express leaf photosynthesis, which is affected by various environmental factors [11]. The FvCB model represents leaf-level biochemical mechanisms, which are the most well-known reactions for photosynthesis, and has been used consistently in various studies [12]. The main assumption of the FvCB model is that the absorbed photosynthetic active radiation affects the photosynthetic capacity of each canopy position and contributes to the overall canopy photosynthesis [13,14]. In addition, to simplify the calculation, the models assume that the vertical distribution of light interception has a negative exponential pattern from the top to the bottom of the canopy [15,16]. However, in these models, the spatial and temporal heterogeneity of light interception are not considered. Many models of canopy photosynthesis have been devoted to reflect the structural characteristics of crops and the scattering of light within the crop canopy [17,18,19]. However, photosynthesis models that explain canopy photosynthesis have a disadvantage in that the model formulas are complicated because of sunlit and shaded leaves. Moreover, while the FvCB model has been used for the analysis of carbon fluxes in an ecosystem, it has rarely been used for the analysis of light intensity and photosynthesis of a greenhouse crop canopy [20]. Therefore, using a simple leaf photosynthesis model and reflecting the changes along the leaf position is suitable for expressing the whole plant photosynthetic rate of the crop [21,22]. Thus, it is necessary to study the method of using specific values for intercepted light intensity in the canopy for simple leaf photosynthesis models.

Preconditions for analyzing accurate light distribution, spatial leaf photosynthetic rate, and whole plant photosynthetic rate of crop canopy are required [23]. From this aspect, construction of the three-dimensional (3D) plant model should reflect the exact physical characteristics of the plant architecture. Ray-tracing is a reasonable approach to integrate optical characteristics such as reflectance and transmittance of leaves and other structures into light simulations. Recently, there have been many studies investigating light distribution in crop leaves using 3D plant models and ray-tracing techniques [7,24,25,26].

To investigate the whole plant photosynthetic rate of mangoes under controlled light intensity, it is necessary to investigate the changes in light interception under artificial and natural lights. Estimating canopy photosynthetic rates by combining the above methods and photosynthesis models is a useful tool in determining strategies for greenhouse crop production. The objectives of this study were to analyze accurate intercepted light intensity using a 3D plant model and ray-tracing method, determine the spatial distributions of the photosynthetic rate, and estimate and validate the whole plant photosynthetic rate of mango under artificial and natural lights in greenhouses.

## 2. Results

### 2.1. Actual Distribution of Intercepted Light Intensity and Measured Whole Plant Photosynthetic Rate

**Figure 1:**
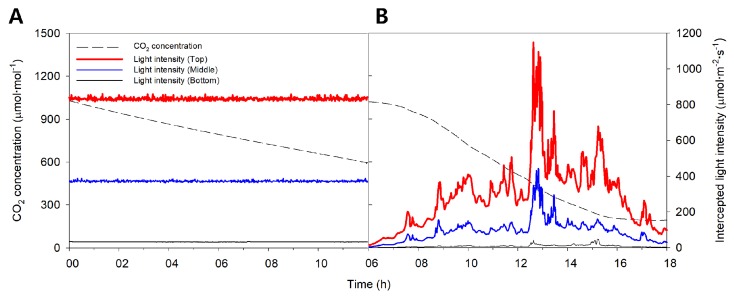
Changes in measured CO_2_ concentration in the closed chamber and intercepted light intensity at the top, middle and bottom of the mango tree on 15 July 2016 under artificial light (**A**) and 21 July 2016 under natural light (**B**) for 12 h.

**Figure 2:**
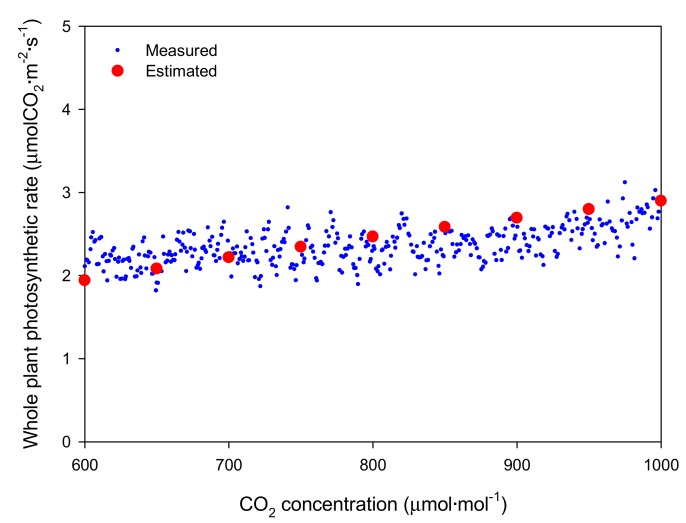
Comparison of measured and estimated whole plant photosynthetic rates of the mango tree according to CO_2_ concentration under artificial light for 12 h.

Under the artificial light, the light intensity at the top of the mango tree was more than twice as large as the middle and bottom positions (Figure 1A). Specifically, the measured intercepted light intensities were 833.1 ± 5.10, 373.0 ± 2.73, and 33.7 ± 0.46 μmol∙m^−2^∙s^−1^ at the top, middle, and bottom, respectively. Fine noise was generated, but the intercepted light intensity was kept at a substantially constant level during the measurement period. The CO_2_ concentration in the closed chamber exponentially decreased from an initial 1000 μmol·mol^−1^ concentration to 600 μmol·mol^−1^ after 12 h. The decrease in CO_2_ concentration over time was calculated, and the whole plant photosynthetic rate of mango was estimated according to CO_2_ concentration (Figure 2). When the CO_2_ concentration inside the chamber was 600 μmol·mol^−1^, the whole plant photosynthetic rate was around 2.0 μmolCO_2_·m^−2^·s^−1^. The whole plant photosynthetic rate did not increase at a CO_2_ concentration of 800 to 900 μmol·mol^−1^, but gradually increased other CO_2_ concentrations.

**Figure 3:**
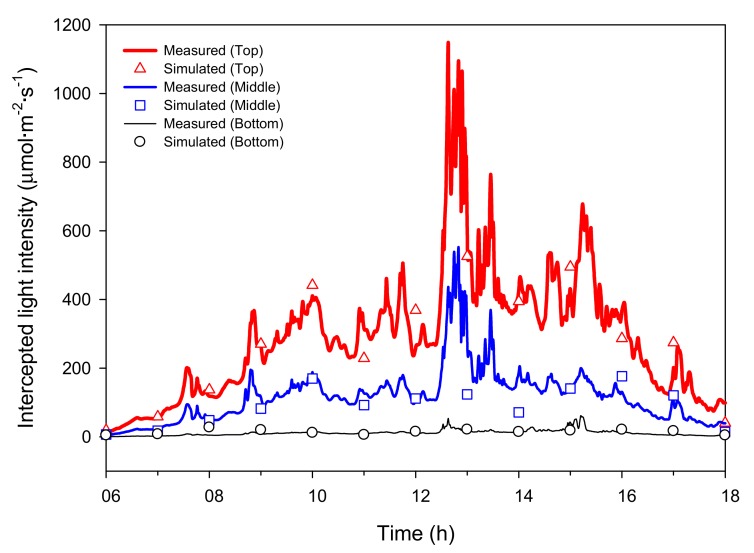
Comparison of measured and simulated intercepted light intensity at the top, middle, and bottom of the mango tree under natural light on 21 July 2016.

**Figure 4:**
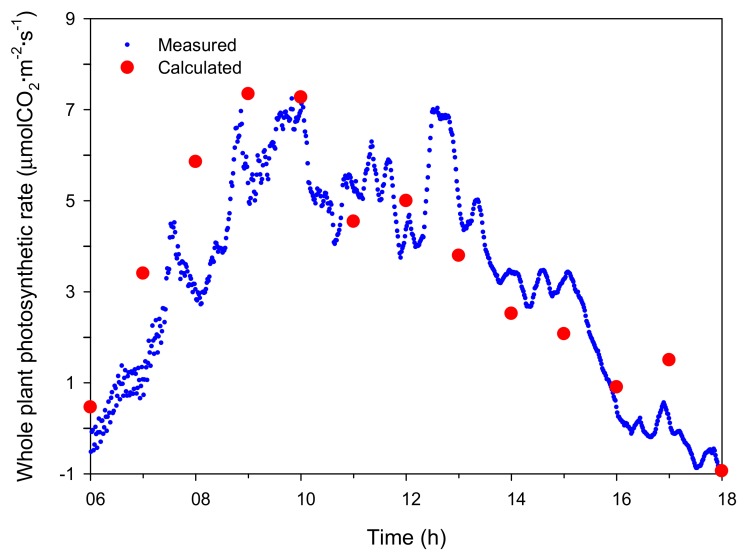
Comparison of measured and estimated whole plant photosynthetic rate of the mango tree under natural light on 21 July 2016.

Under natural light, the intercepted light intensities at the top, middle, and bottom showed rapid changes during the measurement period and showed a maximum value at around 13:00 (Figure 1B and Figure 3). The maximum intercepted light intensities were 1143.9, 548.9, and 57.2 μmol·m^−2^·s^−1^ at the top, middle, and bottom, respectively. The CO_2_ concentration in the closed chamber decreased resembling a sigmoidal curve from the initial 1000 μmol·mol^−1^ concentration to 200 μmol·mol^−1^ after 12 h. The decrease in CO_2_ concentration over time was calculated, and the whole plant photosynthetic rate of the mango was estimated (Figure 4). The whole plant photosynthetic rate started at −0.2 μmolCO_2_·m^−2^·s^−1^ at 06:00, reached its maximum value of 7.0 μmolCO_2_·m^−2^·s^−1^ around 10:00, and then gradually decreased to −1.0 μmolCO_2_·m^−2^·s^−1^ at 18:00.

### 2.2. Simulated Distribution of Intercepted Light Intensity and Estimated Whole Plant Photosynthetic Rate

**Figure 5:**
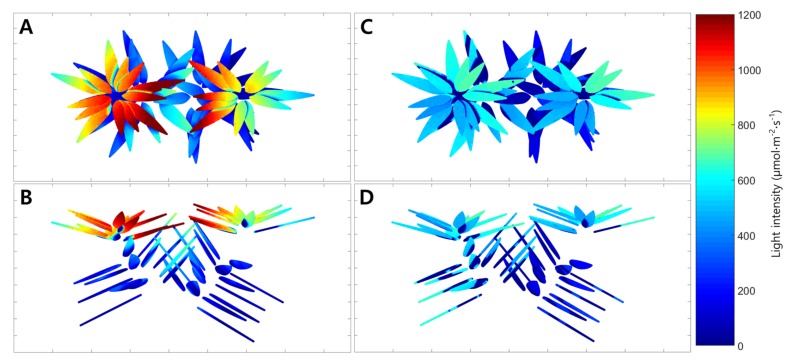
Estimated spatial distributions of light interceptions of the mango tree under artificial light ((**A**), top view; (**B**), front view) and under natural light on 21 July 2016 ((**C**), top view; (**D**), front view).

**Figure 6:**
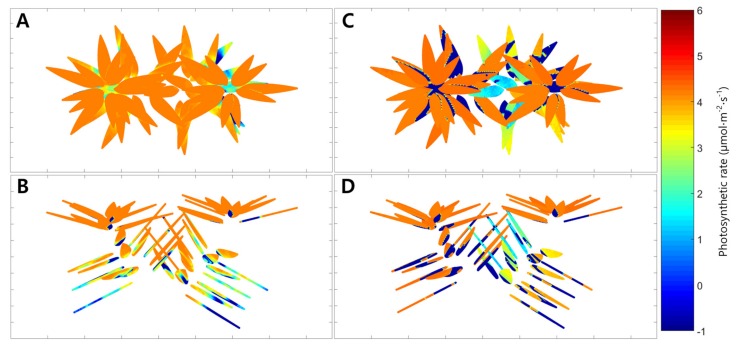
Estimated spatial leaf photosynthetic rates of the mango tree under artificial light ((**A**), top view; (**B**), front view) and under natural light on 21 July 2016 ((**C**), top view; (**D**), front view).

The average simulated intercepted light intensities under the artificial light were 820, 375, and 40 μmol·m^−2^·s^−1^ at the top, middle, and bottom, respectively. When the light distribution of the entire plant was simulated, it was confirmed that the intercepted light intensity was different on each leaf (Figure 5A,B). The maximum intercepted light intensity at the top leaves was 1200 μmol·m^−2^·s^−1^, while the bottom intensity was almost 0 μmol·m^−2^·s^−1^. The distribution of the whole plant photosynthetic rate under the artificial light was expressed in 3D space using the simulated intercepted light intensity and the leaf photosynthetic rate model at a CO_2_ concentration of 500 μmol·mol^−1^ (Figure 6A,B). The photosynthetic rates of the top and middle leaves were expressed as a value of about 4 μmolCO_2_·m^−2^·s^−1^ by the intercepted light intensity above the saturation point. Photosynthetic rates below 0 μmolCO_2_·m^−2^·s^−1^ were observed in some leaves at the bottom. The whole plant photosynthetic rate showed an increasing tendency at CO_2_ concentrations from 600 to 1000 μmol·mol^−1^, which was similar to the actual results (Figure 2). The estimated whole plant photosynthetic rate under the artificial light increased from 2.0 to 2.9 μmolCO_2_·m^−2^·s^−1^ with increasing CO_2_ concentration.

Similarly, the simulated intercepted light intensities at the top, middle, and bottom of the plant under natural light fluctuated like the actual measurements (Figure 3). At 06:00, the intercepted light intensities at the leaves in the top, middle, and bottom began at 0 μmol·m^−2^·s^−1^, but differences among the positions appeared as time passed. At all positions, the maximum values of the simulated intercepted light intensity were observed at 13:00, and the changing tendency in intercepted light intensity was similar to that measured. Using the 3D plant model, the distribution of the intercepted light intensity under natural light was expressed in 3D space at 12:00 on 21 July 2016 (Figure 5C,D). The maximum intercepted light intensities at the top and bottom leaves were 600 and near 0 μmol·m^−2^·s^−1^, respectively. The distribution of the whole plant photosynthetic rate under the natural light at 12:00 on 21 July 2016 was expressed in 3D space using the simulated intercepted light intensity, measured CO_2_ concentration, and leaf photosynthetic rate model (Figure 6C,D). In most of the top leaves, the photosynthetic rate was 4 μmolCO_2_·m^−2^·s^−1^, and these leaves showed the intercepted light intensity exceeded the saturation point. However, unlike under the artificial light, discontinuous sections were observed where the photosynthetic rate was 0 μmolCO_2_·m^−2^·s^−1^ at the middle and bottom leaves. In order to compare with the actual results, whole plant photosynthetic rates were calculated at one hour intervals on 21 July 2016 using the simulated intercepted light intensities, CO_2_ concentrations inside the chamber, and the leaf photosynthetic rate model (Figure 4). The whole plant photosynthetic rate started at 0.2 μmolCO_2_·m^−2^·s^−1^ at 06:00, reached the maximum value of 7.3 μmolCO_2_·m^−2^·s^−1^ at 09:00, and then gradually decreased to −1.0 μmolCO_2_·m^−2^·s^−1^ at 18:00.

### 2.3. Validation of the Intercepted Light Intensity and Whole Plant Photosynthetic Rate

**Figure 7:**
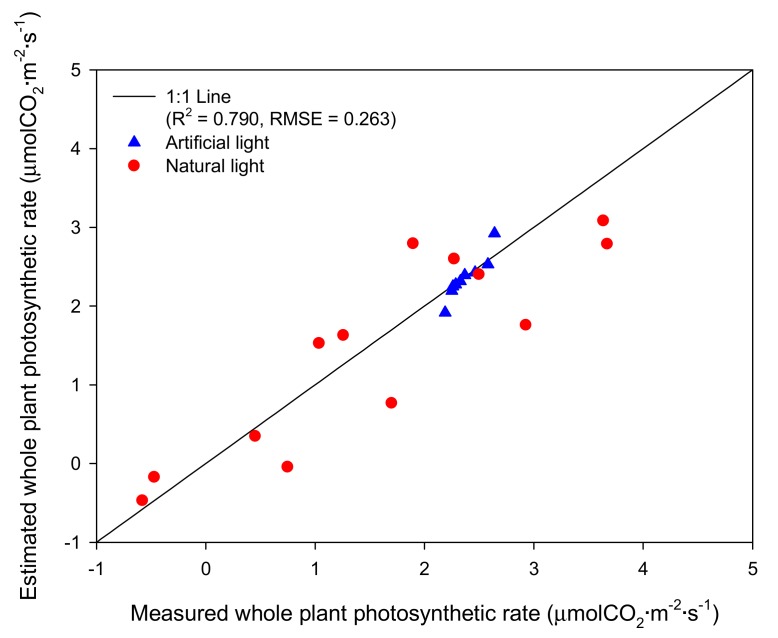
Validation of the measured and estimated whole plant photosynthetic rates of the mango tree under the artificial and natural lights.

The results of validation of the measured and estimated whole plant photosynthesis rates showed that both artificial light and natural light conditions showed linear relationships (Figure 7). The coefficient of determination (*R*^2^) and root mean square error (RMSE) were 0.728 and 0.020 under the artificial light and were 0.715 and 0.447 under the natural light, respectively. At combined light conditions, *R*^2^ was 0.790 and RMSE was 0.263.

## 3. Discussion

Plant photosynthesis is a complicated physiological phenomenon caused by the complex action of various environmental factors. Despite a number of photosynthesis studies conducted at the leaf level, few studies have estimated the photosynthetic rate at the whole plant or canopy level. At the whole plant level, various factors such as intercepted light and the optical and physiological properties related to photosynthetic capacity should be considered, including leaf age, leaf acclimation to light, and nitrogen distribution [26]. In particular, intercepted light intensity is sensitively affected by leaf shape, leaf angle, and plant position inside the canopy architecture [27,28]. Therefore, it is technically difficult to accurately measure and estimate the light intensity reaching the leaf surface of a plant. In this situation, the ray-tracing simulation technique is a good tool to predict the intercepted light intensity at crop leaves on the whole plant level. This can also be of great help in estimating whole plant photosynthetic rates.

The ray-tracing simulation predicted a similar level of intercepted light intensity compared to the actual light intensity measured using sensors mounted on the mango tree (Figure 1). This result implies that ray-tracing simulation is a suitable method for predicting the intercepted light intensity over the crop canopy. However, there was a larger difference between the measured and predicted light intensities under the natural light condition than the artificial light condition. This is probably due to the influence of the black cloth used in the artificial light condition. Compared to the artificial light, there were some effects of scattered light by the greenhouse and chamber under natural light. The intercepted light intensity measured under natural light appeared more homogeneous throughout the crop (Figure 5C,D). As scattered light easily penetrates into the crop canopy [13], scattered light in the greenhouse structure should be considered in simulations for accurate estimation.

Simulation results showed that the whole plant photosynthetic rate increases in the form of a saturation curve with increasing CO_2_ concentration under the artificial light. This occurs because the increase in whole plant photosynthetic rate by CO_2_ concentration followed the increase in leaf photosynthetic rate even though the light intensity at each leaf is different. However, the measured whole plant photosynthetic rate did not appear as a saturation curve (Figure 2). In order to measure the whole plant photosynthetic rate under the artificial light, we measured the change in CO_2_ concentration in the chamber for 12 h (Figure 1A). In this case, the CO_2_ concentration was between 700 and 1000 μmol·mol^−1^ during the nighttime from 19:00 to 05:00. The crop adapts according to the photoperiod and performs photosynthesis in the daytime and translocation action to move assimilation products in the daytime or respiration at night [29]. Therefore, the measured whole plant photosynthetic rate at night may differ from the actual value.

**Figure 8:**
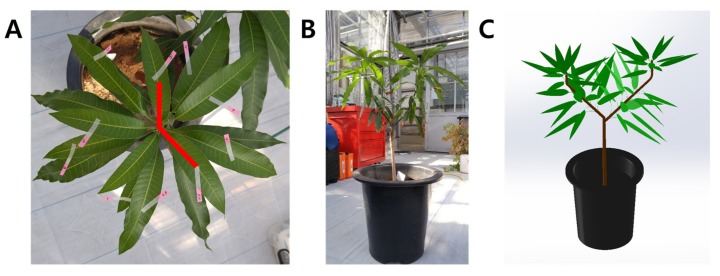
Measurement method of the angle between each mango leaf for estimating phyllotaxis (**A**) and the actual mango tree shape (**B**), and a constructed 3D plant model (**C**).

There are some things to consider in this experiment for more accurate estimation of the intercepted light intensity and whole plant photosynthetic rate. To precisely determine the photosynthetic rate in a closed photosynthetic measurement system, air distribution should be uniform without leakage [30,31]. In addition, the 3D crop model should be similar to the actual crop shape (Figure 8). We constructed the crop shape by examining the leaf form and phyllotaxis of the mango; L-system can be another choice for constructing 3D models [32]. In addition, there are considerations regarding the model equations for calculating whole plant photosynthetic rates. We used a simple form of Equation (3), which includes light intensity and CO_2_ concentration. Stomatal opening and closing changes with time in a day and leaf temperature [33,34]. Accuracy can therefore be reduced when calculating photosynthetic rate in a model that does not reflect stomatal conductivity or leaf temperature. Since the mango used in the experiment was two years old, we used the model assuming that it was the same as a new-born leaf in the previous study [21]. However, it has been reported that leaves present in the lower part of the canopy show a tendency to decrease Rubisco content and RuBP reproduction capacity in various crops [35,36]. For this reason, leaves at the lower part of the canopy decrease photosynthetic ability and nitrogen content inside the tissue. As a result, leaves in shaded areas cannot perform enough photosynthesis even at elevated CO_2_ concentration [37]. Recently, photosynthetic models related to nitrogen distribution have been developed [38] and are necessary to predict more accurate whole plant photosynthetic rates.

Fruit vegetables or fruit trees, such as mangoes, are often pruned to improve fruit quality. By using the simulation performed in this study, it can be useful to evaluate the effect of pruning because the distributions of light intensity and leaf photosynthetic rate vary over the canopy. Because the photosynthetic rate affects fruit yield, management of photosynthesis distribution over the crop canopy is an essential technique for improving crop yield and quality [39]. In addition, efficient CO_2_ fertilization is possible considering that the leaf photosynthetic rate varies depending on the leaf position in the plant. Therefore, this study will contribute to the development of efficient cultivation methods not only for mangoes but also for various fruit crops. By combining light-tracing computer simulation and molecular science, a new photosynthesis study is possible to analyze the light interception and growth of crops.

## 4. Materials and Methods

### 4.1. Measurement of Photosynthetic Rates of Irwin Mango

**Figure 9:**
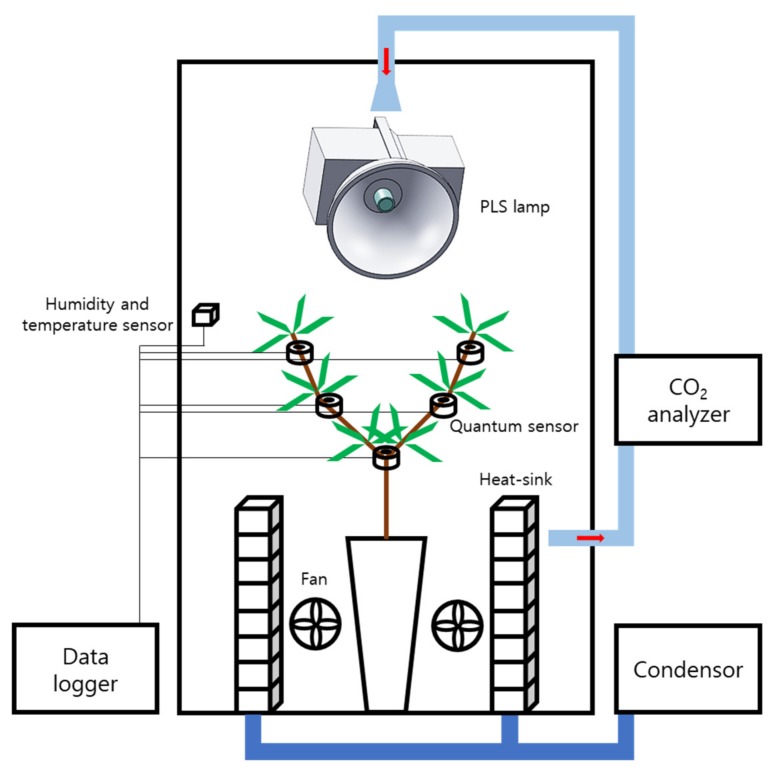
A schematic diagram of the closed chamber for measuring CO_2_ consumption of the mango tree. Under the artificial light, a black cloth was installed outside the chamber and a plasma lamp was used. PLS: plasma lamp.

Whole plant photosynthetic rates were measured using a closed polycarbonate chamber (1 × 1 × 2 m) at the experimental farm of the Seoul National University in Suwon, Korea (37°27′ N, 126°98′ E). A two-year-old Irwin mango (*Mangifera indica* L. cv. Irwin) cultivated in a cylindrical pot (height 40 cm, diameter 30 cm) was placed in the center of the chamber floor (Figure 9). The temperature inside the closed chamber was maintained at 32 °C and the relative humidity was maintained at 60% to 70%. Two radiators circulating cool water were placed along each sidewall to maintain the internal temperature. Two fans were used to mix the internal air. Light intensities at five points of the mango canopy were measured using five quantum sensors (SQ-110, Apogee Instruments, Logan, UT, USA). Two sensors were placed at both the top and middle of the mango canopy, and one sensor was placed at the bottom. Sensor positions were arranged vertically, separated by 15 cm. Changes in CO_2_ concentration inside the chamber were measured using an infrared gas analyzer (LI-840A, LI-COR, Lincoln, NE, USA). Light intensity and CO_2_ concentration were collected by a data logger (GL840, Graphtec, Yokohama, Japan). To measure the chamber leakage, CO_2_ concentration change was measured over an initial CO_2_ concentration of 1300 μmol∙mol^−1^ for 48 h.

To measure the whole plant photosynthetic rate under artificial light, a plasma lamp (PLS, G3, LG electronics Inc., Seoul, Korea) was installed inside the chamber (Figure 9). The lamp position was 1.5 m above the mango canopy. A black cloth was wrapped around the outside of the chamber to block external light. The initial CO_2_ concentration was injected at 1000 μmol·mol^−1^ (Figure 1A). The light intensities and change in CO_2_ concentration inside the chamber were measured for 12 h on 15 July 2016. Similarly, the whole plant photosynthetic rate under natural light was measured for 12 h on 21 July 2016. The artificial light and black cloth were not installed when measuring the whole plant photosynthetic rate under natural light. The initial CO_2_ concentration under natural light conditions was set at 1000 μmol·mol^−1^ (Figure 1B). Changes in light intensity and CO_2_ concentration in the chamber were collected on every seconds. The decrease in CO_2_ concentration was used for the calculation of whole plant photosynthetic rate. At a situation where CO_2_ concentration was decreasing in the chamber, a whole plant photosynthetic rate at a specific CO_2_ concentration was calculated using an average decrease in CO_2_ concentration for 3 min.

### 4.2. Construction of the 3D Plant Model

A 3D plant model was constructed in the same shape as the mango tree to measure the photosynthetic rate. Considerations before making the 3D plant model were as follows: (i) Leaf area and petiole length; (ii) phyllotaxis of the leaves; and (iii) branch pattern, stem length, and diameter. Since the mango leaves used in the experiment cannot be destroyed, leaf area (*LA*) was calculated using a model consisting of leaf length (*L*) and width (*W*): *LA* = −14.623 + 8.074*W* + 0.085*L*^2^ + 0.452*W*^2^ (*R*^2^ = 0.971) [40]. The leaf length, leaf width, and petiole length were measured for all leaves using a ruler. The total *LA* of the mango used in the experiment was 4194.84 cm^2^. To determine the phyllotaxis of the mango leaves, photographs were taken vertically at the apex of the stem and the angle between leaves were measured (Figure 8A and Table 1). In order to create a 3D plant model, the leaves were arranged in the same order as verticillation using the average angle shown in Table 1. The shape of a two-year-old mango branch had a simple form with a new branch growing at the tip of the main stem in a large Y-shape. The stem length, stem diameter, and number of leaves attached to each branch were reflected in the model. The 3D plant model was constructed using measured leaf length and width, phyllotaxis, branch pattern, stem length, and stem diameter. The 3D plant model was developed using 3D CAD software (SolidWorks, Dassault Systemes, Velizy-Villacoublay, France).

### 4.3. Simulation of the Intercepted Light Intensity

Using the 3D plant model, light interception was simulated with ray-tracing software (OptisWorks, OPTIS Inc., Toulon, France). The chamber was designed with the 3D CAD software and assembled with the 3D plant model. Using simulation software, microclimate parameters such as sun direction (coordinates, date, time, zenith, and north direction) and sunlight properties (ratio of direct and diffuse lights), and material parameters such as optical properties of the mango leaves, closed chamber, and greenhouse structure were entered. Optical properties such as transmittance and reflectance of the mango leaves and branches were measured using an integrating sphere (IC2, StellarNet Inc., Tampa, FL, USA) with a spectrometer (BLUE-Wave, StellarNet Inc.) and light source (SL1 Tungsten Halogen, StellarNet Inc.). These values were entered in the preferences section for the leaves in the simulation software. The optical properties of the mango leaves showed little difference in the vertical position within the mango. Ray-tracing simulations were conducted with 1 giga rays and the number of max impacts was set to 10 for all conditions. Detectors were set at 5 mm intervals on all leaf surfaces of the 3D plant model. The intercepted light intensity, transmittance, and reflectance at the detector position on the leaf were calculated through simulation. To simulate the distribution of light interception under artificial light, a modeled artificial lamp was assembled with the 3D plant model in the same position as the actual measurement. The transmittance and reflectance of the closed chamber were set at 0% as the chamber was covered with a black cloth. Similarly, simulation of the distribution of intercepted light intensity under natural light was conducted for 12 h on 21 July 2016. Unlike under artificial light, the black cloth around the chamber was not installed for natural light. The intercepted light intensity was simulated at the top, middle, and bottom of the plant under artificial and natural light. Each position was determined to be the same as where the actual quantum sensors were installed.

### 4.4. Calculation of the Whole Plant Photosynthetic Rate

Since the photosynthetic rate according to the CO_2_ concentration and light intensity was shown as a saturation curve, a simple saturation curve equation was used to determine the photosynthesis model. In the case of ‘Kensington’ mangoes, photosynthetic rate increased with increasing CO_2_ concentration and saturated at a light intensity of 1200 μmol·m^−2^·s^−1^ [22,41]. In addition, Chamchaiyaporn et al. [42] used the rectangular hyperbola equation to express the photosynthetic rate increase with increasing light intensity. Therefore, the two most widely used empirical equations for saturation are summarized as follows. Equations (1) and (2) show the rectangular hyperbola and negative exponential models, respectively.

(1) $$P=\;\frac{a*X}{X+b}+c$$

(2) $$P=a*\left(1-e^{-b*X}\right)+c$$

where P is the net leaf photosynthetic rate (μmolCO_2_·m^−2^·s^−1^), X is the photosynthetic photon flux density (PPFD; μmol·m^−2^·s^−1^) or the CO_2_ concentration (μmol·mol^−1^), and a, b, and c are regression parameters. The two-variable model expressed as the product of the two models has the advantage that it is easy to use because of its simple form [43]. According to Jung et al. [21], a negative exponential model equation is more suitable for expressing the photosynthetic rate in Irwin mangoes. In this study, a leaf photosynthetic rate model for Irwin mangoes suggested by the previous study [21] was used as Equation (3).

(3) $$P=12.928*\left(1-e^{-0.014*I}\right)*\left(1-e^{-0.001*C}\right)-0.889$$

where P is the net leaf photosynthetic rate (μmolCO_2_·m^−2^·s^−1^), I is the photosynthetic photon flux density (PPFD; μmol·m^−2^·s^−1^), and C is the CO_2_ concentration (μmol·mol^−1^). The intercepted light intensity obtained from the ray-tracing simulation and CO_2_ concentration was substituted into the above equation to calculate the photosynthetic rate distribution in the whole plant.

### 4.5. Validation of the Intercepted Light Intensity and Whole Plant Photosynthetic Rate

**Figure 10:**
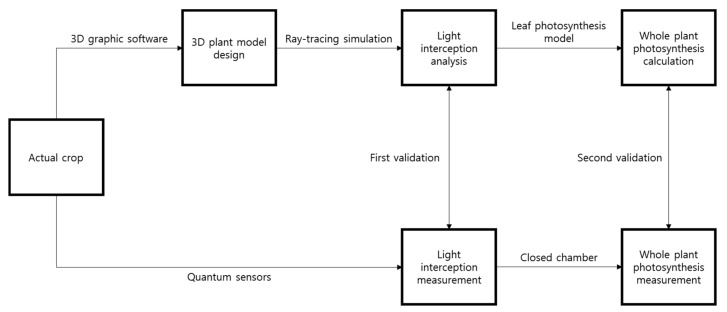
A workflow for the construction of 3D plant model, calculation, and validation of a whole plant photosynthetic rate of the mango tree.

The validation process was performed in two steps. First, the measured light interception at each position in the mango was compared with the estimated interception. Unlike artificial light, natural light intensity changes with time; therefore, the simulated estimated light intensities were compared with the measured intensities with time. Secondly, the measured whole plant photosynthetic rate in the closed chamber was compared with the calculated one using the leaf photosynthetic rate model and simulated intercepted light intensity. A workflow for the construction of the 3D plant model, calculation, and validation of the whole plant photosynthetic rate is described in Figure 10.

## 5. Conclusions

In the case of fruit crops in which the canopy develops in complicated forms, the analysis of light intensity inside the canopy is very difficult but possible through 3D plant modelling and ray-tracing simulation. The light distributions in the canopy estimated by the simulation were similar to those actually measured by quantum sensors. The photosynthetic rates of the whole mango tree calculated using the leaf photosynthetic model with ray-traced light intensities showed good agreement with the measured rates. Through estimating the leaf photosynthetic rates at each position of the tree, efficient pruning and training processes as well as CO_2_ fertilization are possible, which can contribute to improving the productivity and quality of crops grown in greenhouses.

## Figures and Tables

**Table 1:** Measured angles between the mango leaves.

Leaf Number	Angle (°)
1–2	151.5 ± 4.75
2–3	133.2 ± 5.20
3–4	141.5 ± 11.80
4–5	157.6 ± 7.05
5–6	129.9 ± 0.25
6–7	122.1 ± 0.50
7–8	138.0 ± 6.75
8–9	138.8 ± 3.00
9–10	124.4 ± 10.25
10–11	150.9 ± 2.15
11–12	125.9 ± 18.40
Average	137.6 ± 13.38
